# Climate change and environmental health in undergraduate health degrees in Latin America

**DOI:** 10.11606/s1518-8787.2021055002891

**Published:** 2021-04-08

**Authors:** Yasna K Palmeiro-Silva, María Teresa Ferrada, Jorge Ramírez Flores, Ignacio Silva Santa Cruz

**Affiliations:** I University College London Institute for Global Health London UK University College London. Institute for Global Health. London, UK; II Universidad de los Andes Chile Universidad de los Andes, Chile. Escuela de Enfermería. Santiago, Chile; Escuela de Enfermería Santiago Chile; III Investigadora independiente Santiago Chile Investigadora independiente. Santiago, Chile; IV Universidad de Chile Escuela de Salud Pública Santiago Chile Universidad de Chile. Escuela de Salud Pública. Santiago, Chile

**Keywords:** Climate Change, Higher Education Institutions, Schools, Health Occupations, Curriculum, Latin America

## Abstract

**OBJECTIVE:**

Analyze the incorporation of climate change and environmental health courses in the curriculum grids of Medicine, Nursing, Nutrition and Clinical Psychology undergraduate courses in Latin American universities.

**METHODS:**

Descriptive and cross-sectional document review. Curriculum grids of the top ten Latin American universities were analyzed according to the rankings of QS Latin American University 2020, Times Higher Education World University 2020 and Academic Ranking of World Universities 2019. The presence of courses related to climate change and environmental health was sought in each curriculum grid.

**RESULTS:**

104 of the 161 universities included in the study offered Medicine courses, 93 Nursing courses, 77 Nutrition courses and 118 Clinical Psychology courses. Most of the curriculum grids incorporated courses in public health and/or epidemiology (more than 70%); however, between 22% and 41% included courses on environmental health, and only one curriculum grid had a course on climate change in Medicine and Nursing (1%).

**CONCLUSIONS:**

Courses on climate change and environmental health have been scarcely introduced in the curriculum grids of the health field in Latin American universities. This could weaken the important role that health professionals play in providing health care to the population.

## INTRODUCTION

The Covid-19 pandemic has confirmed the global dimension of human health by demonstrating the close interdependence of health with the environment and ecosystems. Similarly, environmental degradation and climate change have become one of the greatest global threats to human health^1^. More specifically in Latin America, changes in precipitation, temperature and extreme weather events have been associated with an increase in morbidity and mortality due to infectious diseases, heat waves and floods^2^^,^^3^. It affects the development of communities and, consequently, public health in the countries of the region, which must also face additional challenges such as poverty and social inequality^4^.

In this scenario, health professionals (HP) play a key role in addressing the threats of climate change on the health of the population, whether in the promotion of healthy environments, in the prevention of unwanted impacts of climate change and the care of people affected through direct work with the population, or in the incidence in public policies of adaptation and mitigation^5^. In general, doctors and nurses provide direct care to people affected by heat waves, floods and contagious diseases such as malaria^6^. Nutritionists ensure proper nutrition of the population and safe food systems, which could be seriously affected by the effects of climate change^7^. Finally, psychologists play a key role in the mental health care of people affected by the effects of climate change, who have high levels of anxiety, depression and post-traumatic stress disorder^8^.

All this demand in the health care of the population requires HP trained in the topics of “climate change” and “environmental health.” However, this training in Latin America is highly variable and it is not clear how they have been inserted in the curriculum grids. This variability and inaction at the educational level could be associated with inaction at the global level with respect to climate change^9^, as well as a general lack of knowledge about the subject and its consideration as something “*new.*” Another cause could be the inexistence of a clear and specific graduate profile that includes competencies and skills in environmental or climate health, which, in turn, could be related to the fact that quality regulatory agencies do not demand clear standards in these areas^10^. In Latin America, unfortunately, there is not enough evidence to establish clear causes.

This deficit of training in the field of climate change and environmental health could compromise the role of HP in protecting the population from the effects of climate change and, consequently, the health of the population. In order to clarify the situation in Latin America, this study analyzed the incorporation of climate change and environmental health courses in the curriculum grids of Medicine, Nursing, Nutrition and clinical psychology undergraduate courses in Latin American universities.

## METHODS

This is a descriptive and cross-sectional document review that analyzes public information available on the websites of Latin American universities in 2020.

For each Latin American country, the top ten universities were selected according to the following rankings independently of the area: QS Latin American University 2020^11^, Times Higher Education World University 2020^12^ and Academic Ranking of World Universities 2019^13^. The final selection of universities considered all the universities that appeared in the three rankings, which were equally attributed to each author of this study. Between April and May 2020, the curriculum grids of the undergraduate programs in Medicine, Nursing, Nutrition and Clinical Psychology were reviewed through the web pages of the institutions. In each curriculum grid, the presence of courses related to “climate change” and “environmental health” was sought, as well as information or other courses potentially related to these topics. All the information was collected by means of a standard formulary, which was later reviewed by an author of the study.

## RESULTS

A total of 161 Latin American universities were included in the study. In the case of Argentina, Brazil, Chile, Colombia, Ecuador, Mexico and Peru, ten or more universities were included. While Bolivia, Guatemala, Honduras, Nicaragua, Paraguay, Puerto Rico and Uruguay five or fewer universities were included because there were no other universities that appeared in the rankings used.

A total of 104 universities offered Medicine course, 93 offered Nursing, 77 Nutrition and 118 Clinical Psychology. Most of the medical programs have a duration between 6 and 7 years; for Nursing, Nutrition and Psychology the duration ranges from 4 to 5 years. 37 curriculum grids could not be obtained, as they were not available on the web pages.

Most of the curriculum grids in Medicine, Nursing and Nutrition incorporate public health and/or epidemiology courses (more than 70%). However, the percentage of environmental health courses is lower (between 22% and 41%), and only one course on climate change has been incorporated as an elective subject in Medicine and Nursing. Table 1 shows the distribution of universities that explicitly incorporate climate change and environmental health courses in the curricula of the health degrees analyzed. The Figure summarizes the number of universities and courses included for each Latin American country.

**Table 1:** Distribution of curriculum grids analyzed that incorporate courses on climate change and environmental health.

	Is there a course on climate change in the curriculum grid?		Is there an environmental health course in the curriculum grid?	
	Yes n (%)	No n (%)	Yes n (%)	No n (%)
Medicine (n= 104)	1 (0.96)	103 (99.04)	38 (36.54)	66 (63.46)
Nursing(n= 93)	1 (1.08)	92 (98.92)	21 (22.58)	72 (77.42)
Nutrition(n= 77)	0 (0)	77 (100)	32 (41.56)	45 (58.44)
Psychology (n= 118)	0 (0)	118 (100)	27 (22.88)	91 (77.12)

**Figure:**
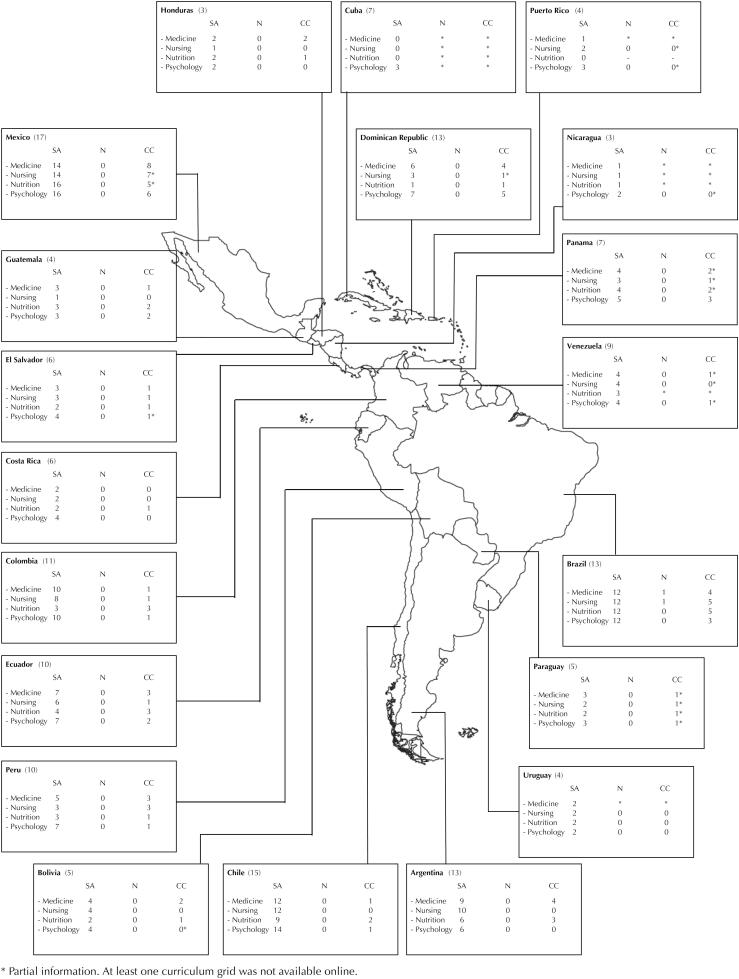
Number of universities (n) per country in Latin America and courses on climate change (CC) and environmental health (SA – salud ambiental) by health degree (N).

## DISCUSSION

This is the first study that identifies the scarce incorporation of climate change and environmental health courses in the curriculum grids of undergraduate Medicine, Nursing, Nutrition and Clinical Psychology courses in Latin American universities. About 1% of the curriculum grids of health degrees have explicitly incorporated a course on climate change, and 30%, on average, have integrated a course on environmental health. These results highlight the insufficient training of HP in these subjects. We must remember that almost 25% of disease is attributed to environmental factors^14^; therefore, we must expand the classic biomedical approach to health towards a more comprehensive approach, which includes environmental or ecological determinants of health, as well as other social determinants.

Climate change and environmental degradation have important consequences on the health of the population. Heat waves increase cardiovascular morbidity and mortality among the most vulnerable people and alter the well-being of the population^15^. A decrease in food production and quality (e.g. grains) negatively affects human nutrition^16^. Extreme weather events such as floods and droughts affect people's mental health^17^. Finally, the closer proximity between humans and wildlife increases the risk of zoonoses^18^ such as the Covid-19 pandemic we are currently experiencing. In these examples and in general, HP play a key role in the prevention, mitigation and response to global environmental and climate change, promoting health protection at different levels through direct care to affected people, as well as better health policies. Thus, the training of future HP in this area not only prepares them as citizens aware of the global changes we are living today, but also becomes an opportunity to achieve the universal health coverage proposed by the World Health Organization (WHO)^19^ and the Sustainable Development Goals^20^.

Our study reports less inclusion of courses on climate change than reported globally. The WHO, in 2019, found that 12 out of 101 countries (11.9%) had developed a national curriculum to train HP on the health impacts of climate change and 27 were under development^21^. On the other hand, the Global Consortium on Climate Change and Health Education, which only includes 6 Latin American institutions, found that 14.28% (12/84) of the Medical schools and 4.76% (4/84) of the Nursing schools that responded to the survey offer education on climate health. This is a kind of education that possibly discusses climate change and health^22^ but might not involve a specific course about these subjects. Consequently, these results could include isolated lessons in a public health or epidemiology course and overestimate the perception of climate change training. In 2019, Mantilla and Li showed that climate change and health issues were scarcely incorporated into medical curricula and strategic planning by Colombian universities^23^.

Based on the current health consequences of global environmental changes, as well as the recognition of the role that ecosystems play in human health, different approaches have been proposed to promote the incorporation of climate change and environmental health topics in the curricula of health degree. Approaches such as “planetary health” and “one health” aim to promote awareness that human health depends on the health of the environment as well as other social determinants of health^24–26^. Complementarily, different strategies, competencies and skills have been proposed to strengthen the training of HP in climate change and environmental health. Specifically, training in knowledge, ethical aspects and social responsibility in relation to these issues is promoted, as well as training in the application of this knowledge in decision-making and strengthening of leadership capacities to be able to influence systems of health and public policies both nationals and internationals^27–31^.

The training of HP based on “planetary health,” “one health,” including ecology and sustainability in health, would allow them to be prepared to face current and future challenges in clinical practice, as well as actively advocate for sustainable development policies that promote social equity based on healthy environments, community participation, promotion of resilience and sustainable, smart and resilient health systems. Although these issues could overload the curriculum grids of degrees in the health area, we must consider that the education and training of HP are dynamic processes that are adapted according to the needs, knowledge and existing technologies in the world. Much of the environmental health courses are in the first semesters of university studies, which could reflect a general introduction to this topic, but still needs to be strengthened. Scientific evidence in relation to global climate and environmental change shows that today there is a clear need to incorporate this issue in the training of future health professionals.

In Latin America, there are different university initiatives that promote education in sustainable development, such as the Sustainable Campus Network (Red Campus Sustentable) in Chile^32^, the Environmental Network of Sustainable Universities (Red Ambiental de Universidades Sostenibles) in Colombia^33^ and the Interuniversity Environmental Network (Red Ambiental Interuniversitaria) in Peru^34^. These networks undoubtedly strengthen and promote the training of students in topics related to the environment and sustainability. In addition to advocating for them to be part of the educational project of higher education institutions, which provides a framework for the development of these areas in the medium and long term. On the other hand, academics and students play a key role in incorporating the topics of climate change, environmental health, and sustainable development in the curriculum grids. Academics can incorporate various theoretical-practical activities promoting training and discussion among students, in addition to producing scientific evidence that is the basis for better decision-making in relation to the insertion of these topics. Some international examples can be observed in the United States^35^^,^^36^ and in Europe, where NurSus TOOLKIT has been developed, an online platform available in seven languages that offers training to future health professionals regarding studies on climate change and sustainability^37^. Finally, the articulation between university networks, academics and researchers, as well as the active participation of students, could strengthen the development of educational standards guides for integration and training on issues of climate change, environmental health and sustainable development, such as it is possible to observe in Australia^38^ and in the United Kingdom^39^.

## LIMITATIONS

This study was based on the official information published by the universities on their respective web pages, which should be considered when analyzing the results. Unfortunately, we were unable to access all the detailed programs of the courses offered that could potentially be related to environmental health (e.g. public health and epidemiology); therefore, in some cases there could be an underestimation of the report. On the other hand, some degrees presented the possibility that students could choose an elective course on climate change or environmental health, but we also did not have access to all the offer of these courses in each degree and university, the results could be underrepresenting this topic because not all elective courses are published.

Some of these limitations could be overcome if universities standardized the description of the programs, but also the availability of this information would have a positive impact on future students, since they would be able to make better decisions regarding their education.

## CONCLUSION

Based on the findings of this study, the role of health professionals in the face of climate change and environmental degradation could be jeopardized due to a lack of training in these areas. Latin America currently requires health professionals who advocate for the care of human health and the environment, understanding the close interdependence that exists between the two topics. Finally, we must extend the concept of health and well-being, advocating for sustainable development in the region, since it is an opportunity to protect and promote a healthy and positive development of society, nature and the economy, without compromising the development of the future generations.
