# Supplementary material for: Climate change and environmental health in undergraduate health degrees in Latin America
Source: Rev Saude Publica. 2021 Apr 8;55:17. doi: 10.11606/s1518-8787.2021055002891 (PMC8032326; doi:10.11606/s1518-8787.2021055002891)
Supplement: Translation

# Translation: Cambio climático y salud ambiental en carreras de salud de grado en Latinoamérica

**Authors:** Yasna K Palmeiro-Silva, María Teresa Ferrada, Jorge Ramírez Flores, Ignacio Silva Santa Cruz

**Affiliations:** I London UK University College London. Institute for Global Health. London, UK; II Chile Universidad de los Andes, Chile. Escuela de Enfermería. Santiago, Chile; Santiago Chile; III Santiago Chile Investigadora independiente. Santiago, Chile; IV Santiago Chile Universidad de Chile. Escuela de Salud Pública. Santiago, Chile

**Keywords:** Cambio Climático, Personal de Salud, Curriculum, Enseñanza, Salud Ambiental, América Latina

## Abstract

**OBJETIVO:**

Analizar la incorporación de cursos de cambio climático y salud ambiental en las mallas curriculares del grado en Medicina, Enfermería, Nutrición y Psicología clínica en universidades latinoamericanas.

**MÉTODOS:**

Revisión documental de tipo descriptiva y transversal. Se analizaron las mallas curriculares de las diez primeras universidades latinoamericanas según los rankings *QS Latin American University 2020, Times Higher Education World University 2020* y *Academic Ranking of World Universities 2019*. En cada malla curricular se buscó la presencia de cursos relacionados al cambio climático y la salud ambiental.

**RESULTADOS:**

De las 161 universidades que se incluyeron en el estudio, 104 ofrecían la carrera de Medicina, 93 de Enfermería, 77 de Nutrición y 118 de Psicología clínica. La mayor parte de las mallas curriculares incorporaron cursos de salud pública y/o epidemiología (más del 70%), sin embargo, entre el 22% y el 41% incluyeron cursos de salud ambiental, y solo una malla curricular tuvo un curso en cambio climático en Medicina y Enfermería (1%).

**CONCLUSIONES:**

Los cursos de cambio climático y salud ambiental han sido introducidos, de forma escasa, en las mallas curriculares del campo de la salud en universidades latinoamericanas. Esto podría debilitar el importante rol que cumplen los profesionales de la salud en la asistencia sanitaria a la población.

## INTRODUCCIÓN

La pandemia del Covid-19 ha confirmado la dimensión global de la salud humana al demostrar la estrecha interdependencia de la salud con el medioambiente y los ecosistemas. De modo similar, la degradación medioambiental y el cambio climático se han convertido en una de las amenazas globales más grandes a la salud humana^1^. Más específicamente en Latinoamérica, los cambios en la precipitación, de temperatura y los eventos climáticos extremos se han asociado a un aumento de la morbimortalidad por enfermedades infecciosas, olas de calor e inundaciones^2^^,^^3^, lo que afecta el desarrollo de comunidades y, en consecuencia, la salud pública de los países de la región, que además deben enfrentar desafíos adicionales como la pobreza y la inequidad social^4^.

Ante este escenario, los profesionales de la salud (PS) juegan un rol clave para enfrentar las amenazas del cambio climático sobre la salud de la población, ya sea en la promoción de medioambientes saludables, en la prevención de impactos no deseados derivados del cambio climático y el cuidado a las personas afectadas por medio del trabajo directo con la población, o en la incidencia en políticas públicas de adaptación y mitigación^5^. De manera general, los/las médicos/as y enfermeros/as otorgan un cuidado directo a personas afectadas por olas de calor, inundaciones y enfermedades transmisibles como la malaria^6^. Los/las nutricionistas velan por una nutrición apropiada de la población y sistemas alimentarios seguros, lo que podría verse seriamente afectado por los efectos del cambio climático^7^. Finalmente, los/las psicólogos/as desempeñan un rol clave en el cuidado de la salud mental de personas afectadas por los efectos del cambio climático, quienes presentan altos niveles de ansiedad, depresión y estrés postraumático^8^.

Toda esta demanda en el cuidado a la salud de la población requiere PS formados en la temática de “cambio climático” y “salud ambiental”, sin embargo, esta formación en Latinoamérica es altamente variable y no se tiene claro cómo se han insertado en las mallas curriculares. Esta variabilidad e inacción a nivel educacional podría asociarse con la inacción a nivel global ante el cambio climático^9^, así como un desconocimiento general del tema y su consideración aún como “nuevo”. Otra causa podría ser la no existencia de un perfil de egreso claro y específico que incluya competencias y habilidades en salud ambiental o climática, lo que, a su vez, podría estar relacionado a que las agencias reguladoras de calidad no exigen estándares claros en estas áreas^10^. En Latinoamérica no existe, lamentablemente, suficiente evidencia para poder establecer causas claras.

Esta falta de formación en el campo de cambio climático y salud ambiental podría poner en jaque el rol que tienen los PS en relación a la protección de la población ante los efectos del cambio climático y, en consecuencia, comprometer la salud de la población. Con el objetivo de clarificar la situación en Latinoamérica, este estudio analizó la incorporación de cursos de cambio climático y salud ambiental en las mallas curriculares del grado en Medicina, Enfermería, Nutrición y Psicología clínica en universidades latinoamericanas.

## MÉTODOS

Esta es una revisión documental de tipo descriptiva y transversal que analiza información pública disponible en las páginas web de universidades latinoamericanas en el año 2020.

Para cada país latinoamericano, se seleccionaron las diez primeras universidades según los siguientes rankings independiente del área: *QS Latin American University 2020*^11^, *Times Higher Education World University 2020*^12^ y *Academic Ranking of World Universities 2019*^13^. La selección final de las universidades consideró todas las universidades que figuraban en los tres rankings, las que fueron equitativamente asignadas a cada autor de este estudio. Entre abril y mayo 2020, se revisaron las mallas curriculares de las carreras de grado en Medicina, Enfermería, Nutrición y Psicología clínica por medio de las páginas web de cada institución. En cada malla curricular se buscó la presencia de cursos relacionados a “cambio climático” y “salud ambiental”, así como información u otros cursos potencialmente relacionados a las temáticas. Toda la información se recolectó por medio de un formulario estándar, la que posteriormente fue revisada por un autor del estudio.

## RESULTADOS

Un total de 161 universidades latinoamericanas fueron incluidas en el estudio. En el caso de Argentina, Brasil, Chile, Colombia, Ecuador, México y Perú se incluyeron diez o más universidades, mientras que Bolivia, Guatemala, Honduras, Nicaragua, Paraguay, Puerto Rico y Uruguay se incluyeron cinco o menos universidades debido a que no existían más universidades que figuraban en los rankings utilizados.

Del total de universidades, 104 ofrecían las carreras de Medicina, 93 de Enfermería, 77 de Nutrición y 118 de Psicología clínica. La mayor parte de las carreras de Medicina tienen una duración entre 6 y 7 años; para Enfermería, Nutrición y Psicología la duración va desde 4 hasta 5 años. Hubo 37 mallas curriculares que no pudieron obtenerse ya que no estaban disponibles en las páginas web.

La mayor parte de las mallas curriculares en Medicina, Enfermería y Nutrición incorporan cursos de salud pública y/o epidemiología (más del 70%). Sin embargo, el porcentaje de incorporación de cursos de salud ambiental es menor (entre un 22% y un 41%), además se ha sido incorporado solo un curso sobre cambio climático como asignatura electiva en Medicina y en Enfermería. La Tabla 1 muestra la distribución de universidades que incorporan explícitamente cursos de cambio climático y salud ambiental en las mallas de las carreras de salud analizadas. La Figura sintetiza el número de universidades y cursos incluidos por cada país latinoamericano.

**Tabla 1:** Distribución de mallas curriculares analizadas que incorporan cursos de cambio climático y salud ambiental.

	¿Existe un curso de cambio climático en la malla curricular?		¿Existe un curso de salud ambiental en la malla curricular?	
	Sí n (%)	No n (%)	Sí n (%)	No n (%)
Medicina (n= 104)	1 (0.96)	103 (99.04)	38 (36.54)	66 (63.46)
Enfermería (n= 93)	1 (1.08)	92 (98.92)	21 (22.58)	72 (77.42)
Nutrición (n= 77)	0 (0)	77 (100)	32 (41.56)	45 (58.44)
Psicología (n= 118)	0 (0)	118 (100)	27 (22.88)	91 (77.12)

**Figura:** Número de universidades (n) por país en Latinoamérica y cursos de cambio climático (CC) y salud ambiental (SA) por carreras de la salud (N).

## DISCUSIÓN

Hasta el momento este es el primer estudio que identifica la escasa incorporación de cursos de cambio climático y salud ambiental en las mallas curriculares de grado en las carreras de Medicina, Enfermería, Nutrición y Psicología clínica en universidades latinoamericanas. Cerca de un 1% de las mallas curriculares de carreras de la salud ha incorporado explícitamente un curso de cambio climático, y un 30%, en promedio, ha integrado un curso de salud ambiental. Estos resultados llaman la atención sobre la insuficiente formación de los futuros PS en esta temática, ya que debemos recordar que casi un 25% de la carga de enfermedades son atribuidas a factores medioambientales^14^, por lo tanto, debemos expandir la clásica aproximación biomédica de la salud hacia una aproximación más integral, que incluya determinantes medioambientales o ecológicos de la salud, así como otros determinantes sociales.

En particular, el cambio climático y la degradación medioambiental tienen importantes consecuencias sobre la salud de la población. Por ejemplo, las olas de calor aumentan la morbimortalidad cardiovascular entre las personas más vulnerables y alteran el bienestar de la población^15^; una disminución en la producción y calidad de alimentos (p.ej. granos) impacta negativamente la nutrición humana^16^; los eventos climáticos extremos como inundaciones y sequías afectan la salud mental de las personas^17^; y por último, la mayor cercanía entre los humanos y la vida animal salvaje aumenta el riesgo de zoonosis^18^ como lo es la pandemia de Covid-19 que estamos viviendo. En estos ejemplos y de manera general, los PS juegan un rol clave en la prevención, mitigación y respuesta ante el cambio climático y ambiental global, abogando por la protección de la salud en diferentes niveles mediante un cuidado directo a las personas afectadas, así como por mejores políticas sanitarias. En este sentido, la formación de futuros PS en esta temática no solo los prepara como ciudadanos conscientes de los cambios globales que vivimos hoy en día, sino que se convierte en una oportunidad para lograr la cobertura sanitaria universal propuesta por la Organización Mundial de la Salud (OMS)^19^ y los Objetivos de Desarrollo Sostenible^20^.

Nuestro estudio reporta menor inclusión de cursos sobre cambio climático que lo reportado a nivel mundial. La OMS en 2019 publicó que 12 de 101 países encuestados (11,9%) habían desarrollado un currículum nacional para entrenar a PS sobre los impactos en salud del cambio climático y 27 se encontraban bajo desarrollo^21^. Por otra parte, el Consorcio Global sobre Cambio Climático y Educación en Salud, que solo incluye 6 instituciones latinoamericanas, encontró que un 14,28% (12/84) de las escuelas de Medicina y un 4,76% (4/84) de las escuelas de Enfermería, que respondieron la encuesta, ofrecen educación sobre salud climática, educación que posiblemente analice cambio climático y salud^22^, pero que no implica un curso específico en el tema. Consecuentemente, estos resultados podrían incluir lecciones aisladas en algún curso de salud pública o epidemiología y sobre estimar la percepción de formación en cambio climático. En 2019, Mantilla y Li evidenciaron que la temática de cambio climático y salud ha sido incorporada escasamente en los currículos de Medicina al igual que en las planificaciones estratégicas por parte de las universidades colombianas^23^.

Con base en las consecuencias sanitarias actuales que provienen de los cambios ambientales globales, así como el reconocimiento del rol que juegan los ecosistemas en la salud humana, se han propuesto diferentes aproximaciones que promueven la incorporación de temáticas sobre el cambio climático y la salud ambiental en los planes de estudio de las carreras de la salud. Las aproximaciones como las de “planetary health” y “one health” tienen como objetivo promover la conciencia de que la salud humana depende de la salud del medioambiente y también de otros determinantes sociales de la salud^24–26^. De modo complementario, se han propuesto diferentes estrategias, competencias y habilidades para fortalecer la formación de los PS en la temática de cambio climático y salud ambiental. En específico, se promueve la formación en conocimiento, aspectos éticos y de responsabilidad social en relación con estos temas, así como el entrenamiento en la aplicación de ese conocimiento en la toma de decisión y fortalecimiento de capacidades de liderazgo para poder influir sobre los sistemas de salud y las políticas públicas tanto nacionales como internacionales^27–31^.

La formación de los PS basada en “*planetary health*”, “*one health*”, incluidos los elementos de ecología y sostenibilidad en salud, les permitiría prepararse para enfrentar los retos actuales y futuros en la práctica clínica, así como advocar activamente por políticas de desarrollo sostenible, que promuevan equidad social a partir de medioambientes saludables, participación comunitaria, promoción de resiliencia y sistemas de salud sostenibles, inteligentes y resilientes. Si bien estos temas podrían sobrecargar las mallas curriculares de las carreras en el área de la salud, debemos considerar que la formación y el entrenamiento de los PS son procesos dinámicos que se adaptan de acuerdo con las necesidades, conocimiento y tecnologías existentes en el mundo. Cabe destacar que gran parte de los cursos de salud ambiental se encuentran en los primeros semestres de la formación universitaria, lo que podría reflejar una introducción general a este tema, pero aún necesita fortalecerse. La evidencia científica en relación con el cambio climático y ambiental global muestra que hoy existe la clara necesidad de incorporar esta temática en la formación de los futuros profesionales de la salud.

En Latinoamérica, existen diferentes iniciativas universitarias que promueven la educación en desarrollo sostenible como la Red Campus Sustentable en Chile^32^, la Red Ambiental de Universidades Sostenibles en Colombia^33^ y la Red Ambiental Interuniversitaria en Perú^34^. Estas redes, sin duda, fortalecen y promueven la formación de estudiantes en las temáticas relacionadas al medioambiente y la sostenibilidad. Además de abogar para que sean parte del proyecto educativo de las instituciones de educación superior, lo que otorga un marco de desarrollo de estas áreas a mediano y largo plazos. Por otra parte, los académicos y estudiantes desempeñan un rol clave en la incorporación de los temas de cambio climático, salud ambiental y desarrollo sostenible en las mallas curriculares. Los académicos pueden incorporar diversas actividades teórico-prácticas que favorezcan la formación y discusión entre los estudiantes, además de producir evidencia científica que sea base para una mejor toma de decisión en relación con la inserción de esos temas. Algunos ejemplos internacionales se pueden observar en Estados Unidos^35^^,^^36^ y en Europa, donde se ha desarrollado NurSus TOOLKIT, una plataforma on line disponible en siete idiomas que ofrece capacitación a futuros profesionales de la salud en cuanto a estudios sobre el cambio climático y la sostenibilidad^37^. Finalmente, la articulación entre redes universitarias, académicos e investigadores, así como la activa participación de los estudiantes podría fortalecer el desarrollo de guías de estándares educativas para la integración y formación en temas sobre el cambio climático, la salud ambiental y el desarrollo sostenible, como es posible de observar en Australia^38^ y en Reino Unido^39^.

## LIMITACIONES

Este estudio se basó en la información oficial publicada por las universidades en sus respectivas páginas web, que se debe tomar en consideración al momento de analizar los resultados. Lamentablemente no pudimos tener acceso a todos los programas detallados de los cursos ofertados que potencialmente podrían estar relacionados a la salud ambiental (p. ej. salud pública y epidemiología), por lo tanto, en algunos casos pudiese existir una subestimación del reporte. Por otra parte, algunas carreras presentaban la posibilidad de que los alumnos pudieran realizar un curso electivo sobre cambio climático o salud ambiental, pero tampoco tuvimos acceso a toda la oferta de esos cursos en cada carrera y universidad, por lo que los resultados podrían estar subpresentando esta temática debido a que no todos los cursos electivos son publicados.

Algunas de estas limitaciones podrían ser superadas si las universidades estandarizaran la descripción de los programas de formación, pero también la disponibilidad de esta información tendría un impacto positivo en los futuros estudiantes, ya que serían capaces de tomar mejores decisiones respecto a su formación.

## CONCLUSIÓN

En base a la evidencia encontrada en este estudio, el rol de los profesionales de la salud frente al cambio climático y degradación ambiental podría estar en jaque debido a una carencia de formación en estas áreas. Latinoamérica requiere actualmente a profesionales de la salud que aboguen por el cuidado de la salud humana y el medioambiente, entendiendo la estrecha interdependencia que existe entre ambos. Finalmente, debemos extender la concepción de salud y bienestar, abogando por un desarrollo sustentable en la región, ya que es una oportunidad para proteger y promover un desarrollo saludable y positivo de la sociedad, la naturaleza y la economía, sin comprometer el desarrollo de las generaciones futuras.
